# The effects of an e-health brisk walking intervention in increasing moderate-to-vigorous physical activity in physically inactive older people with cognitive frailty: study protocol for a randomized controlled trial

**DOI:** 10.1186/s13063-023-07335-8

**Published:** 2023-05-17

**Authors:** Rick Yiu Cho Kwan, Justina Yat Wa Liu, Paul Hong Lee, Olive Suk Kan Sin, Julia Sze Wing Wong, Mei Rosemary Fu, Lorna Kwai Ping Suen

**Affiliations:** 1 School of Nursing, Tung Wah College, Hong Kong SAR, China; 2 School of Nursing, The Hong Kong Polytechnic University, Hong Kong SAR, China; 3 Clinical Trials Unit, University of Southampton, Southampton, UK; 4 Board of Director Office, Pok Oi Hospital, Hong Kong SAR, China; 5 School of Nursing, George Washington University, Washington, USA

**Keywords:** Moderate-to-vigorous physical activity (MVPA), E-health, Brisk walking, Cognitive frailty

## Abstract

**Background:**

Cognitive frailty is a risk for many adverse health outcomes that are commonly observed in older people. Physical activity is known to be effective to reserve cognitive frailty but the prevalence of physical inactivity is still high in older people. E-health enhances behavioural change effects through an innovative way to deliver behavioural change methods that can enhance the behavioural change effects. However, its effects on older people with cognitive frailty, its effects compared with conventional behavioural change methods, and the sustainability of the effects are unclear.

**Methods:**

This study employs a single-blinded, two-parallel-group, non-inferiority, randomized controlled trial design with a 1:1 group allocation ratio. Eligible participants are aged 60 years or above, have cognitive frailty and physical inactivity, and possess a smartphone for more than six months. The study will be conducted in community settings. In the intervention group, participants will receive a 2-week brisk-walking training followed by a 12-week e-health intervention. In the control group, participants will receive a 2-week brisk-walking training followed by a 12-week conventional behavioural change intervention. The primary outcome is minutes of moderate-to-vigorous physical activity (MVPA). This study aims to recruit a total of 184 participants. Generalized estimating equations (GEE) will be used to examine the effects of the intervention.

**Ethics and dissemination:**

The trial has been registered at ClinicalTrials.gov (Identifier: NCT05758740) on 7th March 2023, https://clinicaltrials.gov/ct2/show/NCT05758740, and all items come from the World Health Organization Trial Registration Data Set. It has been approved by the Research Ethics Committee of Tung Wah College, Hong Kong (reference number: REC2022136). The findings will be disseminated in peer-reviewed journals and presented at international conferences relevant to the subject fields.

**Trial registration:**

The trial has been registered at ClinicalTrials.gov (Identifier: NCT05758740) and all items come from the World Health Organization Trial Registration Data Set. The latest version of the protocol was published online on 7th March 2023.

## Introduction

### Background and rationale

Cognitive frailty is the co-existence of physical frailty and mild cognitive impairment (MCI) [1]. It is more closely associated with adverse health markers (e.g. depression and malnutrition) than MCI or physical frailty [2]. Cognitive frailty is common in community-dwelling older people with a prevalence rate of 8.7% in Hong Kong [3]. The global prevalence is increasing from 6% during 2012–2017 to 11% during 2018 and 2020 [4]. Cognitive frailty is a risk for many adverse health outcomes, such as dementia, dependency and mortality [5]. However, cognitive frailty is considered to be possibly reversible with effective interventions [6, 7]. The probability of reversibility is higher at an earlier stage, thus timely intervention is essential [8].

Physical activity plays a significant role in reversing cognitive frailty [9]. It is because physical activity breaks the vicious cycle between cognitive frailty and physical inactivity by alleviating the pathological conditions of cognitive frailty (e.g. inflammation, glucose metabolism, sarcopenia, and insulin resistance) [10–12]. Weekly 150 min of moderate-to-vigorous physical activity (MVPA) shows additional benefits compared with sitting and light physical activity [13]. Although the benefits of MVPA are promising, physical inactivity remains common in older people, with a prevalence rate of over 60% in the USA and over 40% in Hong Kong and China [14–16]. Physical inactivity in older people is defined as performing fewer than 150 min of moderate-to-vigorous physical activity (MVPA) per week by the World Health Organization [17]. Prevalence of physical inactivity in older people increases with age and level of cognitive impairment [18, 19]. MVPA can reduce the risk of worsening cognitive frailty and improve cognitive and physical functions [7, 20, 21].

Conventionally, a range of behavioural change techniques, standardized by the Coventry, Aberdeen, and London – Refined (CALO-RE) taxonomy (e.g. goal-setting, self-monitoring) and delivered by human interventionists, have been used to promote physical activity [22]. However, the conventional behavioural change interventions that have proven to be effective with younger people are less effective with older people [23]. E-health provides an alternative delivery method to conventional behavioural change methods delivered by human interventionists [24]. E-health uses persuasive technology methods delivered by computer interventionists that potentially enhance behavioural change effects [25]. Systematic reviews showed that e-health technologies increase physical activity in older people with a large effect [26–30]. Albeit the fact that e-health can more effectively promote desirable behaviours (e.g. MVPA), its effects are unknown in the context of vulnerable population (i.e. older people with cognitive frailty) and sustainability, and in comparison with conventional behavioural change interventions.

### Objectives

This study aims to compare the immediate and sustained effects of an e-health intervention and a conventional behavioural change intervention in older people with cognitive frailty in (1) improving MVPA, (2) reducing cognitive frailty, (3) improving cognitive function, (4) improving physical performance, and (5) improving physical activity motivation.

### Trial design

This study employs a single-blinded, two-parallel-group, non-inferiority, randomized controlled trial.

## Methods

This study trial protocol reports the methods following the Standard protocol Items: Recommendations for Interventional Trial (SPIRIT) 2013 guideline [31].

### Study setting

This study will be conducted in community settings. Participants will be recruited from community centres for older people in Hong Kong, including District Elderly Community Centre (DECC) and Neighbourhood Elderly Centre (NEC). All of the community centres for older people in Hong Kong regularly provide recreational social activities for community-dwelling older people aged 60 years or above [32].

### Eligibility

#### Inclusion criteria

1. Aged 60 years or above;
2. Cognitive frailty, defined as the co-existence of MCI and frailty at either the pre-frail or the frail level [33–35];
3. Physical inactivity, defined as < 150 min of MVPA every week in the 4 weeks preceding the study, as confirmed by a Rapid Assessment of Physical Activity (RAPA) score of ≤ 4 (i.e. regularly underactive) [17, 36]; and
4. Possession of a smartphone on which Samsung Health and WhatsApp can be installed for > 6 months

#### Exclusion criteria

1. Impaired mobility caused by conditions that require medication (e.g. severe arthritis) and inability to walk briskly outdoors, as defined by a modified Functional Ambulatory Classification score of < 7) [37];
2. Depressive symptomatology, as defined by a Geriatric Depression Scale (GDS) score of ≥ 8 [38];
3. Probable dementia, as defined by a Montreal Cognitive Assessment (MoCA) score of < 20 [39];
4. Physically unfit because of chronic illness, as defined by a Physical Activity Readiness Questionnaire (PAR-Q) score of ≥ 1, and certified by a medical doctor as not physically fit to participate in brisk walking [40]; or
5. Enrolled in other interventions to promote physical activity or training.

### Interventions

#### Intervention components

There are three intervention components: (1) brisk walking training, (2) a conventional behavioural change intervention, and (3) an e-health intervention, as shown in Table 1. All of the intervention components will use various behavioural change techniques (BCT), as specified by CALO-RE taxonomy [41].

**Table 1:** Intervention content summary

Component	BCTs	Materials	Procedures	Who	How	Where	Duration	Frequency	Duration
Brisk-walking training	Information provision	BW package	Lecture	RA	F2F	Community centres	1 h	2 times/week	2 weeks
	Demonstration		Demonstration			Outdoor parks			
CBCI	Goal setting Prompts Self-monitor Social support Information provision	CBCI manual	PA counselling	RA	F2F	Community centres	1 h	1 time/week	12 weeks
		PA logbook							
E-health	Goal setting Prompts Self-monitor Social support Information provision	E-health manual	PA counselling	RA	Remote	Self-determine	1 h	1 time/week	12 weeks
		Smartphone		Smartphone					

*BW package* Hong Kong Leisure and Cultural Services Department Brisk Walking Training Programme package, *CBCI* conventional behavioural change intervention, *PA* physical activity, *RA* research assistant, *F2F* face-to-face

##### Brisk walking training

As shown in Table 1, two BCTs are used: (1) information provision and (2) demonstration. We adopt these two BCTs because the Brisk Walking Training Program provided by the Leisure and Cultural Services Department, Hong Kong Government used only these two BCTs [42]. This study follows the contents of this programme to implement brisk walking training. A trained research assistant will deliver face-to-face lectures at the community centres and conduct face-to-face training in outdoor parks proximal to the participants’ homes, such that the participants can practise brisk walking daily. Each training session will last for 1 h and sessions will be conducted twice a week for 2 weeks.

##### Conventional behavioural change intervention

As shown in Table 1, five BCTs are used: (1) goal setting, (2) self-monitoring, (3) prompts, (4) social support, and (5) information provision. The conventional behavioural change intervention employing these five BCTs is based on our pilot study that it shows favourable outcomes on the amount of MVPA, cognitive function, frailty and physical functions [6]. This study follows the physical activity counselling contents (i.e. motivational interviewing, regular social support) using the five BCTs to implement the conventional behavioural change intervention following a standardized procedural manual used in the pilot study [6]. Physical activity logbooks will be used to record and monitor the participants’ physical activity performance and levels. A trained research assistant will deliver group-based conventional behavioural change intervention face-to-face at the community centre with groups of 5–8 participants. Each session will last for 1 h and will be conducted once a week for 12 weeks.

##### E-health

As shown in Table 1, the same set of BCTs as in the conventional behavioural change intervention is used. However, the BCTs are delivered using e-health methods. The e-health physical activity counselling (i.e. motivational interviewing, regular social support) is based on the one implemented in our pilot study [6]. A trained research assistant will deliver the e-health physical activity counselling through remote coaching and the participants’ smartphones, which will have Samsung Health and WhatsApp installed to serve as computer interventionists and assist the research assistant in delivering physical activity counselling (i.e. e-health methods) following a standardized procedural manual in the pilot study [6]. Because the e-health physical activity counselling is delivered remotely, the participants can self-determine the place where to attend it. Similar to the conventional behavioural change intervention, each session will last for 1 h and will be conducted once a week for 12 weeks. During these 12 weeks, the primary duty of the research assistant will be to train the participants to uptake the BCTs delivered by the computer interventionists (i.e. smartphones) and their peers on WhatsApp to practise and increase the amount of brisk walking gradually. This aims at fostering sustained engagement and regular participation in MVPA via brisk walking even after the 12-week training period.

Samsung Health is a free and commercially available wearable activity tracker installable in smartphones that continually monitors walking behaviours (e.g. steps, walking speed, walking time, brisk walking time) and physical activity (e.g. number of minutes of MVPA). Wearable activity trackers offer a favourable low-cost tool to promote physical activity as evidenced by systematic reviews [43, 44]. It plays the role of a computer interventionist because it automatically coaches users through *goal setting*, *self-monitoring* and *prompts*. Samsung Health can *set goals* for daily physical activity in a tailored manner based on users’ performance in previous weeks. Samsung Health also automatically logs physical activity behaviours (e.g. brisk walking time) to allow users to *self-monitor* their brisk walking and MVPA. Samsung Health also *prompts* users to perform physical activities to achieve goals in line with their patterns of physical activity.

WhatsApp is a freeware application that allows users to send text messages and voice messages, make voice and video calls, and share images, documents, user locations and other content. It will assist the research assistant in delivering the e-health intervention through *goal setting*, *self-monitoring*, *prompts*, and *social support*. The research assistant will be able to read the participants’ activity records logged by Samsung Health and shared by the participants. *Goal setting* will be tailored to the participant’s individual capabilities. If the participants miss the e-prompts from Samsung Health, the research assistant will *prompt* the participants to attend to the e-prompts and perform physical activities following their goals. The research assistant will summarize the past achievements of the participants and provide feedback on their performance to ensure effective *self-monitoring*. The research assistant will group coach participants with similar capabilities (*n* = 5–8) in a WhatsApp group, thus promoting peer *social support* among the participants.

#### Groups

In the intervention group, participants will first receive the 2-week *brisk-walking training*. In addition, the participants in this group will learn about Samsung Health and WhatsApp. Subsequently, the participants in this group will receive the 12-week *e-health* programme. The intervention period will last for 14 weeks.

In the control group, the participants will first the 2-week *brisk-walking training*. In addition, the participants in this group will learn how to use a physical activity logbook to record their physical activity performance. Subsequently, the participants in this group will receive the *conventional behavioural change intervention* programme for 12 weeks. Finally, the intervention period will last for 14 weeks.

In both groups, participants are free to participate in any other activities provided by the elderly community centres or other organizations. However, participants are prohibited to participate in other additional activities related to brisk-walking training provided by other organizations during the intervention period.

#### Criteria for discontinuing interventions

If actual or potential harms are identified, the trial steering team will report them to the advisory panel, which will decide to consider suspending or terminating the trials.

#### Interventionists

A trained research assistant will deliver the e-health intervention to the intervention group. The same trained interventionist will deliver the conventional behavioural change intervention to participants in the control group. The training will be provided by the principal investigator (i.e. RK) following the corresponding procedural manuals (i.e. e-Health manual, conventional behavioural change intervention manual) used in the pilot study [6].

#### Intervention fidelity

Training will be provided for the interventionist following an intervention protocol checklist that covers the most important steps used in our pilot study by the trial steering team [6]. The interventionist will implement the intervention procedures (e.g. WhatsApp, face-to-face meetings) following the procedure checklist and record their procedures to comply with the intervention protocols. This will ensure that all of the BCTs are delivered as planned. All communication on WhatsApp and via telephones will be logged. The trial steering team will be added to all of the WhatsApp groups as silent participants. The trial steering team will check the WhatsApp communications of a randomly selected group and conduct weekly site visits to observe face-to-face physical activity counselling sessions at a randomly selected centre. These random checks will enable the trial steering team to monitor the interventions implemented by the interventionist and ensure that the interventions are delivered in compliance with the protocol. Regular research team meetings will be conducted to determine whether the interventionist has followed the intervention protocol.

#### Tailoring

The goals of the brisk walking exercise (e.g. time, intensity) will be determined by 1) autosuggestions from Samsung Health based on the participant’s performance in the last week, 2) the participants’ wishes, and 3) suggestions made by the human interventionist based on the participants’ baseline fitness. The goals will be reviewed weekly and progressively increased (i.e. safe weekly increase < 20%/week) based on the joint decisions between the interventionist and the participants until the participants attain the target (i.e. 150 min MVPA/week).

### Outcomes

Demographic data include age, gender, level of education, number of chronic illnesses, financial satisfaction, living arrangement, years of smartphone usage, and perceived ability to use a smartphone. Demographic data will be collected at T0 (i.e. week 0) only. The primary outcome is MVPA. The four secondary outcomes are cognitive frailty, cognitive function, physical function, and physical activity motivation. All outcome data will be collected at T0 (i.e. week 0), T1 (i.e. week 15), and T2 (i.e. week 41).

#### MVPA (primary outcome)

MVPA will be quantified in MVPA minutes measured by a wrist-worn ActiGraph GT3X + , which has good criterion validity in differentiating between MVPA and non-MVPA in older people compared with indirect calorimetry (sens = 0.836, spec = 0.894, AUC = 0.932, *p*< 0.001) [45]. An MVPA minute is defined as a minute in which the ActiGraph mounted on the right wrist records physical movement (i.e. vector magnitude) of above 4212.9 count/min [45]. Only at least 10 min of continuous MVPA will be counted as valid MVPA minutes because the World Health Organization advises that engaging in MVPA for 150 min per week has health benefits when each session lasts for > 10 min [17]. Physical activity will be quantified by valid MVPA minutes measured over 7 consecutive days, as a 7-day interval is sufficient to discern an individual’s pattern of physical activity and is a widely used standard in studies of physical activity [46]. Only MVPA minutes measured on valid days (i.e. ActiGraph wearing time > 10 h/day) for a valid period (i.e. valid days > 3) will be considered valid [47].

#### Cognitive frailty

Cognitive frailty will be measured by a previously validated instrument, which quantifies cognitive frailty by two components: frailty (i.e. robust, pre-frailty, frailty) and MCI (i.e. yes, no) [48]. Frailty will be measured using Fried Frailed Phenotype, which quantifies frailty based on five components: weight loss, exhaustion, low physical activity, slow gait and weakness [33]. FFP scores range from 0 to 5, with one point assigned for the presence of one component. A higher score indicates greater frailty. Those with 0, 1–2 or 3–5 points are classified as robust, pre-frailty or frailty, respectively MCI will be measured using the MoCA score by assigning “yes” to the score ≤ 25 and “no” to the score > 25 [49]. The variable has six levels from level 1 at “no cognitive frailty” (i.e. robust without MCI) to level 6 at “cognitive frailty” (i.e. frailty with MCI). A higher score indicates a greater level of cognitive frailty.

#### Cognitive function

Cognitive function will be measured using MoCA [49], which comprises 30 dichotomous items. One point will be assigned to one correct answer. The total score ranges from 0 to 30. A higher score indicates a higher level of cognitive function. MoCA has strong correlations with other cognitive screening instruments, including Mini-Mental State Examination (*r* = 0.90) and Saint Louis Mental Status Examination (*r*= 0.83) [50]. MoCA also has good validity in detecting MCI (sensitivity = 0.90, specificity = 1.00) [49].

#### Physical function

Walking speed and functional fitness will be measured as forms of physical function. Walking speed will be measured by the Timed Up-and-Go test, which quantifies the total time needed for a participant to stand up from a chair, walk a 3-m distance, walk back to the chair, and sit down [51]. Functional fitness will be measured by the 30-s Chair Stand Test, which measures the number of stands a person can complete in 30 s [52].

#### Physical activity motivation

The Revised Motivation for Physical Activity Measure (RMPAM) will be used to measure physical activity motivation. RMPAM comprises 30 7-point items, each of which has five subscales with a total score ranging from 30 to 210. A higher score indicates greater motivation to perform physical activity [53].

### Participant timeline

**Fig. 1:**
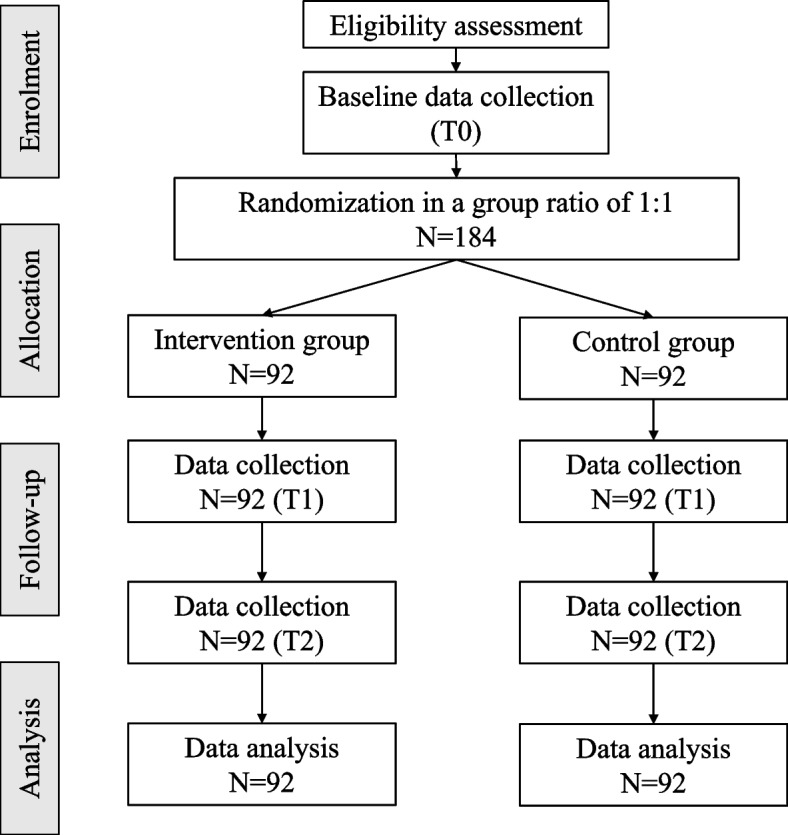
Legend: T0: Baseline for pre-treatment assessment (i.e. week 0), T1: The week immediately after the intervention (i.e. week 15), T2: The week 6 months after the completion of the intervention (i.e. week 41)

As shown in Table 2 and Fig. 1, potential participants will be enrolled on the study in the phase of enrolment. The potential participants will be screened for eligibility. Eligible participants will be invited to sign the written informed consent after being explained the study details. After the enrolment phase, the pre-treatment assessment (T0, i.e. week 0) will commence. Demographic and outcome data will be collected. After that, participants will be randomly allocated to either the intervention or control group. Then, the interventions will commence. In the week immediately after the intervention (T1, i.e. week 15), the post-treatment assessment will commence and outcome data will be collected. Data of all outcome variables will be collected once again in the 6-month (i.e. 26 weeks) follow-up period (T2, i.e. week 41).

**Table 2:** Schedule of enrolment, intervention, and assessments (SPIRIT-Figure)

Timepoint	Enrolment	Pre-treatment assessment	Allocation	Intervention	Post-treatment assessment	6-month follow-up
		T0			T1	T2
Enrolment						
Eligibility screen	x					
Informed consent	x					
Group allocation			x			
Interventions						
E-health				x		
CBCI				x		
Data collection						
Demographic		x				
Outcome						
MVPA		x			x	x
Cognitive frailty		x			x	x
Cognitive function		x			x	x
Physical function		x			x	x
Physical activity motivation		x			x	x

### Sample size

We adopt a priori power analysis using the Web-based software GLIMMPSE and employ a general linear mixed model [54]. We set the level of significance at 0.05, the power at 0.9, the number of repeated measures at 3 (i.e. T0, T1, T2), the number of groups at 2 (i.e. the intervention and control groups) and the allocation ratio between the two groups at 1:1. To estimate the effects, we estimate the immediate effect of the intervention according to the effect on the primary outcome compared between two groups with the same interventions (i.e. e-health vs conventional behavioural change intervention) as reported in the pilot study of this project [55]. We assume the effects to be sustained until the 6-month follow-up. We estimate that the sample size is 132. We assume a cumulative drop-up rate of 18.1% at T1 and T2 [56]. The total sample size estimated is 184 participants, with 92 participants in each group.

### Recruitment

Health talks on e-health, brisk walking, and cognitive frailty will be conducted in community centres for older people. At the end of the talks, participants will be directly recruited. The programme will be introduced to our collaborative organizations. We will produce both printed and electronic promotional materials (e.g. posters, and short videos). With the help of the collaborative organizations, the promotional materials will reach the members of the collaborative organizations through the webpage of the hosting and collaborative organizations, post, email, WhatsApp, and social media (e.g. Facebook, YouTube Channel). The project team and the staff members of the collaborative organizations will help recruit potentially eligible participants. To enhance the recruitment rate, a cognitive frailty symptom checklist and the eligibility criteria will be posted in all the promotional materials. We will announce to all the potential participants and the staff member of the collaborative organizations to read the information and validate if the potential participants fulfil the eligibility criteria before eligibility screening will be conducted on them. This is to ensure that the interested participants are more likely to be eligible to join this study. With these measures, the estimated recruitment rate is expected to be above 50%.

### Assignment of interventions

All participants will be individually randomized into groups. Permuted block randomization will be used to randomly select block sizes and a group ratio of 1:1 will be used. A random allocation sequence list will be generated by the web-based application Research Randomizer [57]. The process will be conducted by an independent statistician, who will not participate in any other parts of the proposed study. The statistician will assign group labels to the participants based on their sequence of entries referring to the random sequence list, thus ensuring that other members of the research team cannot foresee the group allocations [58].

In the proposed study, only the outcome assessor will be blinded to the group labels, as it will be impossible to blind the participants and interventionists. The group labels will not be revealed to the outcome assessor and will not appear on any documents. The participants, family members and staff of the centres will be prohibited from disclosing the group labels of the participants. It will not be possible to blind the participants or the interventionists. To further minimize the risk of contamination in the same centre, the participants will be instructed to not teach the intervention-related e-health skills (e.g. the self-paced brisk walking training function in Samsung Health) to the participants in the control group. The WhatsApp groups will only be managed by the interventionists, who will forbid non-participants from joining.

### Data collection and management

Data will be collected by the project implementation team, including a research assistant who does not know the group label and a group of student helpers after they have completed the training provided by the trial sponsor (RK). To avoid unintentional missing data, a computerized online data collection application Qualtrics (www.qualtrics.com) will be used. Qualtrics will prompt the data collectors for the unfilled data field. Data collectors must provide reasons for leaving the data fields blank before they can submit the data to the server. On the day after the data collection, the research assistant will conduct preliminary data analysis to do the range check to ensure that all data entered are correct. When out-of-range data are identified, the research team will investigate the causes and do the rectifications needed (e.g. re-conduct the data collection, and telephone clarifications to the participants). Qualtrics employs many technological methods to ensure the confidentiality of the data (e.g. high-end firewall system, encryption) [59]. The data on Qualitrics will be downloaded and saved on the password-protected cloud server at Tung Wah College for at least seven years according to the requirement of the ethics committee after each round of data collection.

To promote participant retention and complete follow-up, a completion certificate issued by Tung Wah College will be provided to participants who completed the intervention and the follow-up assessment. For participants who discontinue or deviate from intervention protocols (e.g. too low attendance rate), all of their outcome data will also be collected at T1 and T2. A certificate presentation ceremony will be held for completers of the study after the data collection at T2.

All data saved on Qualtrics are coded so that no personal identifiers will be saved on Qualtrics. A code will be assigned to a participant. A codebook keeps the participants’ names and phone numbers. Phone numbers and names are marked on the codebook because we want to make sure that participants with the same name can be identified. The data collector assigns a code to each participant when they are being recruited. The data collector does not participate in data analysis or any other parts of the study. The codebook is saved on a cloud server (i.e. OneDrive, TWC) which is only accessible to the PI. Data will be collected using an online data collection platform (i.e. Qualtrics). In the online data set, only the code is saved, and the data set contains no identifiers. Only the PI have the access to both the codebook on OneDrive TWC and the dataset on Qualtrics. There are no direct links between the code and the participants’ identifiers. Codebook with personal identifiers (i.e. name and telephone number) will be deleted upon the completion of the study.

### Statistical methods

Demographic and outcome data at baseline will be reported either as means with standard deviation or as frequencies with percentages based on their level of measurement. Generalized estimating equations (GEE) will be used to separately test the hypotheses on the five outcomes, which are the dependent variables (i.e. MVPA, cognitive frailty, cognitive function, physical function, and physical activity motivation). The independent variables will be the same across all GEEs: group (two categories: intervention and control groups), timepoint (three categories: T0, T1, T2), and group x timepoint. The primary interpretation of the results will be based on the analysis based on the intention-to-treat principle without adjusting for possible covariates [60, 61]. Sensitivity analyses will be conducted and reported to compare the results of different methods of analysis, such as intention-to-treat and per-protocol analyses [62]. The level of significance will be set at 0.05. Missing data will be managed following Jakobsen’s algorithm according to the missing pattern with various methods (e.g. no imputation, multiple imputations) [63].

### Monitoring

A trial steering team including two academics in the disciplines of nursing (i.e. the author RK, JW) and one social worker who is the in-charge of a group of elderly community centres (i.e. OS) will direct the whole study process. The project implementation team comprising one research assistant and a team of nursing student helpers will be responsible to provide direct support for the trial. An advisory panel comprising four nursing academics (i.e. the author JL, PL, MRF, and LS) specializing in e-health, behavioural change, non-pharmacological intervention trials, and bio-statistics will provide advice to the project and play the role of data monitoring (e.g. quality of data collection procedures, data entry, and data analysis). The trial steering team will run interim analyses regularly and report them to the advisory panel. The trial steering team will meet the project implementation team bi-weekly to monitor the implementation adherence and provide suggestions to solve the implementation problems. The advisory panel is independent of the trial sponsor (i.e. the author RK) and competing interests, the trial steering team, and the project implementation team. The trial steering team reports to the advisory panel that they will meet once per three months to advise on upholding the quality of the trial (e.g. intervention implementation, data collection, and data analysis).

There is no anticipated harm or compensation for trial participation. The project implementation team will meet the participants regularly. If suspected adverse events and other unintended effects of the intervention are reported by the participants, the implementation team will document them on an incident record and report them to the trial steering team. The trial steering team will report them to the advisory panel to decide if they are related to the trial and subsequently make a final decision to respond (e.g. withdraw the affected participants from the trial or terminate the trial). An independent company will be hired to audit the trial and provide an audit report once per year.

### Ethics and dissemination

All participants will be required to sign the written informed consent to participate in the proposed study. The consent will be obtained by the research assistant after explaining the study to the participants according to the information materials. No identifying images or other personal or clinical details of participants are presented here or will be presented in reports of the trial results. The participant information materials and informed consent form are available from the corresponding author on request.

In case there are changes in the protocol, the research team will notify the sponsor, funder, and Research Ethics Committee. Then, the research venues (i.e. centres for older people) will be informed and a copy of the revised protocol will be sent to them. If there are any deviations from the protocol, the changes will be fully documented using a breach report form and the details in the clinical trial registry will also be updated.

After the completion of the study, only the research team members have the right to access the final full dataset.

The findings will be disseminated in peer-reviewed journals and presented at international and local conferences with themes related to this area. The research team complies with the authorship eligibility guideline recommended by the International Committee of Medical Journal Editors [64].

Details of the protocol, participant-level data, and statistical codes are available from the corresponding author on request.

## Discussion

Older people with cognitive frailty are potentially treatable to prevent further adverse health outcomes, the policymakers should put cognitive frailty, this relatively new health marker, a priority to treat in primary healthcare settings. E-health through smartphones is innovative which has great potential in developing effective interventions to promote health behaviours (e.g. physical activity) in older people with cognitive frailty and other health concerns (e.g. dementia) [44, 65]. This e-health brisk walking intervention may provide effective interventions to promote cognitive health prevention and preservation in primary healthcare settings. This project is innovative in fostering collaboration between academic and community settings (e.g. elderly community centres) where used to provide social and recreational activities, to provide potentially effective primary health promotion services. The project will launch an e-health intervention in several community centres for older people. Although there will only be approximately 192 participants in the study, we will continue to promote the adoption of the intervention in different community centres during and after the completion of the proposed study. A training package with written and video materials will be produced to facilitate the adoption of the intervention by other unparticipated elderly community centres. If the intervention proves effective in reducing cognitive frailty, the health of older people is expected to improve as well. Older people will benefit from living more independent lives, having fewer chronic diseases (e.g. dementia) and having better functionality (e.g. better mobility).

### Trial status

Recruitment is expected to begin on the 1st of May 2023 and until the 31st of July 2024.

## Data Availability

The data will be provided upon request.

## Abbreviations

MCI: Mild cognitive impairment
MVPA: Moderate-to-vigorous physical activity
BCT: Behavioural change technique
RAPA: Rapid Assessment of Physical Activity
GDS: Geriatric Depression Scale
MoCA: Montreal Cognitive Assessment
PAR-Q: Physical Activity Readiness Questionnaire
RMPAM: Revised Motivation for Physical Activity Measure
GEE: Generalized estimating equations
